# Nematode-Induced Growth Factors Related to Angiogenesis in Autoimmune Disease Attenuation

**DOI:** 10.3390/life13020321

**Published:** 2023-01-23

**Authors:** Marta Maruszewska-Cheruiyot, Katarzyna Krawczak-Wójcik, Martyna Michniowska, Michael James Stear, Maja Machcińska, Maria Doligalska, Katarzyna Donskow-Łysoniewska

**Affiliations:** 1 Laboratory of Parasitology, General Karol Kaczkowski Military Institute of Hygiene and Epidemiology, 01-163 Warsaw, Poland; 2 Department of Parasitology, Institute of Functional Biology and Ecology, Faculty of Biology, University of Warsaw, 02-096 Warsaw, Poland; 3 Department of Animal, Plant and Soil Science, Agribio, La Trobe University, Bundoora 3086, Australia; 4 Department of Experimental Immunotherapy, Faculty of Medicine, Lazarski University, 02-662 Warsaw, Poland

**Keywords:** experimental autoimmune encephalomyelitis, colitis, growth factors, nematode adaptation, angiogenesis

## Abstract

**Simple Summary:**

Helminths are parasitic worms that influence their host in a variety of ways, including the production of growth factors and the creation of blood vessels (angiogenesis). Parasites are used to control autoimmune diseases and parasite-derived molecules are widely studied for their therapeutic potential. Accordingly, the objective of this study was to evaluate the influence of parasitic nematode infection on growth factors related to angiogenesis in murine colitis and multiple sclerosis. We observed significant changes in both models of autoimmune disorders. In addition, parasitic infection remodeled the creation of vessels in the brains of mice with multiple sclerosis. Nematode-derived factors are promising tools to fight autoimmune diseases and to study angiogenesis.

**Abstract:**

Accumulating data suggest an important role of growth factors in autoimmune diseases and parasitic nematode infections. Nematodes are used in clinical studies of autoimmune diseases and parasite-derived molecules are widely studied for their therapeutic potential in various types of disorders. However, the effect of nematode infection on growth factors in autoimmune disorders has not been studied. The objective of this study was to evaluate the influence of infection with the intestinal nematode *Heligmosomoides polygyrus* in murine autoimmune models on the production of growth factors. Here, the level of a variety of growth factors related mainly to angiogenesis was evaluated by protein array in the intestinal mucosa of C57BL/6 dextran sodium sulfate-induced colitic mice and in cerebral spinal fluid of experimental autoimmune encephalomyelitis (EAE) mice infected with nematodes. In addition, vessel formation was evaluated in the brains of EAE mice infected with *H. polygyrus*. A significant influence of nematode infection on the level of angiogenic factors was observed. Parasitic infection of colitic mice resulted in upregulation of mucosal AREG, EGF, FGF-2, and IGFBP-3 in the intestine of the host and better adaptation (infectivity). In EAE mice, infection increased the level of FGF-2 and FGF-7 in CSF. In addition, remodeling of brain vessels was observed, with a higher density of long vessels. Nematode-derived factors are promising tools to fight autoimmune diseases and to study angiogenesis.

## 1. Introduction

Autoimmune diseases (ADs) are characterized by immune-mediated damage. Growth factors related to angiogenesis can contribute to pathogenesis [1]. Angiogenesis is an important process in the body in normal development and during repair processes, as well as in disease [2]. Angiogenesis is a complex, multistep process including cell proliferation, differentiation, migration and cell–cell interactions, and requires appropriate regulation [3]. Angiogenic processes create new vessels from already functioning ones. Various growth factors are involved in angiogenesis, including vascular endothelial growth factors (VEGF), fibroblast growth factors (FGF), epidermal growth factors (EGF), hepatocyte growth factor (HGF), insulin-like growth factors (IGF), and platelet-derived growth factors (PDGF) [4]. 

Multicellular parasites, including nematodes, have developed multiple immunoregulatory molecules. Consequently, parasites and their products are being tested as treatments for autoimmune diseases [5]. Clinical studies on patients with inflammatory bowel diseases such as ulcerative colitis and Crohn’s disease have shown that nematode infection can reduce inflammation of the intestine and colon [6,7]. Similarly, in patients suffering from multiple sclerosis, a positive outcome of helminth therapy has been observed [8]. The therapeutic effect is mainly correlated with inhibition of the proinflammatory response associated with Th1 and Th17 T cells and induction of regulatory activity of Treg cells [9]. However, the host–parasite relationship is extremely complex, and processes that take place in the host during infection are not well explored. Nematodes need nutrition and to eliminate waste products. The formation of a network of blood vessels by parasites during infection is one of their adaptation strategies [10]. Nematodes can influence angiogenesis by modulating the production of angiogenic factors or by producing molecules that mimic host molecules or homologues of proteins with roles in angiogenesis.

The aim of this study was to evaluate the influence of infection with the intestinal nematode *Heligmosomoides polygyrus* during autoimmune diseases on growth factors related to angiogenesis. Infection with *H. polygyrus* is a model for human infection with *Necator americanus*. This hookworm is a significant cause of disease and is used as a treatment for autoimmune disorders (helminth therapy) in clinical studies [11]. Amphiregulin (AREG), epidermal growth factor (EGF), fibroblast growth factor 2 (FGF-2), FGF-7, granulocyte-macrophage colony-stimulating factor (GM-CSF), hepatocyte growth factor (HGF), insulin-like growth factor binding protein-3 (IGFBP-3), platelet-derived growth factor-AA (PDGF-AA), PDGF-BB, and vascular endothelial growth factor (VEGF) were evaluated in the intestinal mucosa of C57BL/6 colitic mice and in the cerebral spinal fluid (CSF) of EAE mice with *H. polygyrus* infection. In addition, the creation of new vessels in the brains of mice with EAE was evaluated by expression of CD31. Here, we examine angiogenesis in a model with both inflammatory and parasitic disease; a state corresponding to human helminth therapy.

## 2. Materials and Methods

### 2.1. Colitis Model

Eight-week-old pathogen-free C57BL/6 males were allowed to adapt to laboratory environment one week before the experiment began (*n* = 48). Severe colitis was provoked by the administration of 3% dextran sulphate sodium (DSS) (TdB Consultancy AB, Uppsala, Sweden), a 35–50 kDa sulphated polymer, in drinking water; it was administered two days before oral infection with 300 L3 of *H. polygyrus*, and administration of DSS was continued until the end of the experiment. The following clinical symptoms confirmed the successful induction of colitis: reduced body weight, soft stools, fecal bleeding, and diarrhea. The disease activity index (DAI) was calculated as the sum of weight loss compared to baseline weight, stool consistency, and bleeding [12]. The same researcher assessed all indications of colitis, including changes in body weight, stool consistency, and the presence of occult blood in the stools using a paper test (HemoActive, Diagnosis, Białystok, Poland). Mice were divided into four experimental groups. One of the groups received DSS (COL), the second group of mice was infected with *H. polygyrus* (HP), and the third group received both DSS and *H. polygyrus* (HP COL). Uninfected, untreated mice constituted the control group (CTR). The mice were euthanized and samples were obtained six days after infection, when the parasite was in its fourth larval stage. 

### 2.2. Burden of Parasitic Infection

The small intestines of colitic mice were removed, ligated at both ends with cotton twine to prevent digested matter from contaminating the medium, and incubated for two hours at 37 °C in Petri dishes containing RPMI-1640 Medium with L-glutamine (2 mM), penicillin (100 U/mL) and streptomycin (100 µg/mL) (Biowest, Lakewood Ranch, FL, USA). The existence of the bursa at the caudal end of pre-male larvae was used to determine the sex of the L4 stage. The larvae were counted separately in each mouse.

### 2.3. Preparation of Blood, Small Intestine and Colon Mucosa Samples

Blood samples taken after heart puncture from animals with colitis were used to make serum. The small intestine and colon were removed, opened longitudinally, and washed in cold PBS, pH 7.4. Mucosa samples were prepared by scraping the inner layer of the small intestine and colon with a microscope slide. The samples were diluted in 2 mL of cold PBS, pH 7.4, including protease inhibitor cocktail tablets (Roche Applied Science, Indianapolis, IN, USA), mixed with disposable needles of decreasing diameter, then centrifuged at 4000× *g* at 4 °C for 45 min. Prior to cytokine and growth factor analysis, the supernatant was kept at −80 °C.

### 2.4. ELISA

ELISA was used to determinate the levels of IL-1β, IL-6, TNF-α, IL-10, and TGF-β in the serum, small intestine, and colonic mucosa of mice with induced colitis according to the manufacturer’s guidelines (eBiosciences, Thermo Fisher, Waltham, MA, USA). The colorimetric reaction was defined at 450 nm with a Synergy™ H1 Microplate Reader (BioTek, Winooski, VT, USA). The mean optical densities (OD) of triplicate cultures were compared to recombinant cytokine-prepared standard curves.

### 2.5. EAE Model

Eight-week-old pathogen-free C57BL/6 males were allowed to adapt to laboratory environment week before the experiment began (*n* = 48). Experimental autoimmune encephalomyelitis (EAE) was induced by subcutaneous injection in the rear flanks with 200 μg of myelin oligodendrocyte glycoprotein MOG_35–55_ (purity > 95%) per mouse emulsified in complete Freund’s adjuvant (CFA) containing 300 μg of *Mycobacterium tuberculosis* H37RA strain. Immediately afterwards and again 2 days later, the animals received an intraperitoneal injection of 400 ng of *Bordetella pertussis* toxin (PTX; Sigma, St. Louis, MO, USA) in 100 μL of PBS, pH 7.2. Mice were orally infected with 300 L3 of *H. polygyrus* 21 days postimmunization. Clinical signs and ascending paralysis in EAE were assessed as described before [13]. Four experimental groups of mice were formed. The first group was immunized with EAE (EAE), the second group of mice was infected with *H. polygyrus* (HP), and the third group was immunized with EAE and infected with *H. polygyrus* (HP EAE). The control group consisted of uninfected, untreated mice (CTR). The mice were euthanized and samples were obtained six days after infection, when the parasite was in its fourth larval stage.

### 2.6. Cerebral Spinal Fluid (CSF)

Cerebrospinal fluid (CSF) from EAE mice was collected as described before [13]. Briefly, a syringe was gently introduced into the atlanto-occipital membrane between the occipital protuberance and the spine of the atlas. Slow aspiration of CSF produced roughly 20 µL of clear liquid with no blood contamination. The samples were centrifuged at 2000× *g* for 15 min at 4 °C.

### 2.7. Visualization of the Vascular Endothelium

For histological evaluation, the brains of EAE mice were removed and placed in 10% sucrose solution for 24 h. The specimens were subsequently immersed in sucrose solution (Sigma, St. Louis, MO, USA) at escalating concentrations for three days to prepare the tissue for cryosectioning. Sections eight micrometers thick were incubated with primary rat anti-CD31 monoclonal antibody [ER-MP12] (Invitrogen, Thermo Scientific, Waltham, MA, USA) and secondary rabbit anti-rat IgG (Alexa Fluor 594) (Invitrogen, Thermo Scientific). The CD31 protein is a well-characterized marker of angiogenesis. Nikon Eclipse Ti-S microscope images were captured and analyzed with NIS Elements F2.30 software (Nikon, Tokyo, Japan).

### 2.8. Antibody Arrays

Proteins associated with angiogenesis in CSF isolated from mice with induced EAE or in the mucosa of the small intestine isolated from mice with induced colitis were analyzed with the Angiogenesis Array (Proteome Profiler Mouse Angiogenesis Array Kit; R&D Systems, Minneapolis, MN, USA) and Mouse Growth Factor Array (Ray Biotech, Peachtree Corners, GA, USA), respectively, according to the manufacturer’s instructions. The exposure time was 5 min, and the examination took 20 min since chemiluminescence signals degrade over time. The Syngene G-Box was used to scan the membranes, and the signal values were evaluated using Image J software. The array’s internal positive and negative controls were used to normalize the signals.

### 2.9. Statistical Analysis

All experiments and tests were performed in triplicate to ensure reliable results. The significance of differences was defined with Student’s *t* test (two-tailed unpaired) or the Mann–Whitney test. When more than two groups were analyzed, analysis of variance was caried out (one- or two-way ANOVA; GraphPad Software Inc., La Jolla, CA, USA). When the *p*-value was less than 0.05, the ANOVA was followed by post hoc analysis using Tukey’s multiple comparisons method. The data were presented as mean ± SEM or mean ± SD. A *p* value of <0.05 was considered to be statistically significant.

## 3. Results

### 3.1. Symptoms of Colitis

**Figure 1:**
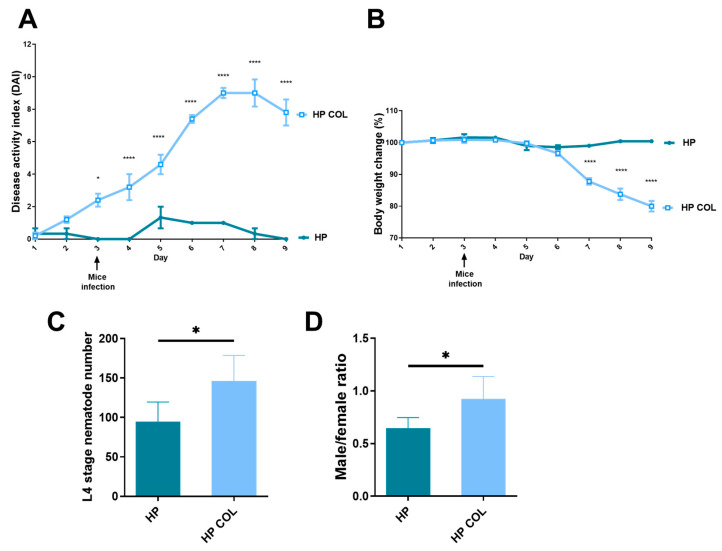
Symptoms of colitis after infection with *H. polygyrus*. C57BL/6 male mice were given 3% dextran sulphate sodium (DSS) in drinking water for two days before being infected orally with 300 L3 *H. polygyrus*. Disease activity index (**A**) was determined using weight change, stool consistency, and blood content. The mice were weighed every day for 9 days until the end of the experiment. Weight change (**B**) is expressed as a percentage of the initial weight. The number of L4 stage nematodes (**C**) was counted for each mouse. Ratio (**D**) was counted as the number of male nematodes divided by the number of female nematodes in each mouse. HP—mice infected with *H. polygyrus*; HP COL—mice with colitis infected with *H. polygyrus*. Data are expressed as mean values ± SEM or SD from one representative of three independent experiments (*n* = 5 mice/group). * *p* < 0.05 and **** *p* < 0.0001.

C57BL/6 mice that received DSS and were infected with *H. polygyrus* developed colitis. Weight was significantly lower from day 7 of the experiment, four days after infection with nematodes. DAI based on weight change, diarrhea, and fecal blood was significantly higher from day 3 of the experiment (1 day before infection with *H. polygyrus*) in comparison with control mice infected with nematodes but without colitis. At the end of the experiment, mice treated with DSS and infected with nematodes had DAI around 8 compared to 0 for the control group (Figure 1A). The weight decreased to around 80% of the starting value, when the control group was around 100% of the original weight (Figure 1B).

### 3.2. Nematodes Adapt to Colitis in C57BL/6 Mice

*H. polygyrus* adapts to the colitis milieu in BALB/c mice strain [13]. To evaluate if colitis promoted adaptation of *H. polygyrus* in C57BL/6 mice, parasites were counted and the sex of the L4 stage was determined for each mouse. The number of L4 stage *H. polygyrus* present in submucosal tissue was significantly enhanced in mice with colitis compared to untreated mice (Figure 1C). The number of pre-male and pre-female parasites was also counted. Similarly, the ratio of male to female was significantly higher in mice with colitis in comparison with control animals (Figure 1D). 

### 3.3. Nematode Adaptation to Colitis Correlate with Changes in Immune Response 

To evaluate how the immune response correlates with adaptation of nematodes to the inflammatory conditions, the level of proinflammatory cytokines (IL-1β, IL-6, TNF-α) and regulatory (TGF-β, IL-10) was evaluated. Treatment of mice with DSS and infection with parasites resulted in increased levels of IL-6, TNF-α, and IL-10 in serum; IL-1β and IL-6 in the colon; and IL-6 in the small intestine. Differences of IL-1β and TGF-β in serum; TNF-α, IL-10, and TGF-β in the colon; and IL-1β, TNF-α, IL-10, and TGF-β in the small intestine were not statistically significant (Table 1).

### 3.4. Colitis Induction and Parasitic Infection Influence Level of Angiogenic Factors in the Intestine

**Figure 2:**
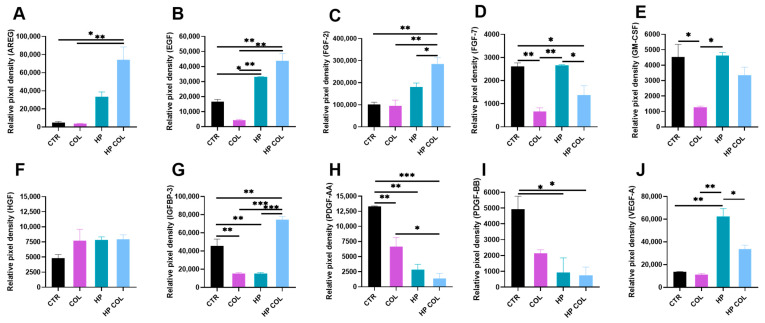
Level of angiogenic factors in the small intestine of C57BL/6 mice with induced colitis and parasitic infection. C57BL/6 male mice were given 3% dextran sulphate sodium (DSS) in drinking water for two days before being infected orally with 300 L3 *H. polygyrus*. Mean relative pixel density of AREG (**A**), EGF (**B**), FGF-2 (**C**), FGF-7 (**D**), GM-CSF (**E**), HGF (**F**), IGFBP-3 (**G**), PDGF-AA (**H**), PDGF-BB (**I**), VEGF-A (**J**). Data are presented as mean pixel density ± SD of two technical replicates. Data are representative of three independent experiments (*n* = 3). * *p* < 0.05, ** *p* < 0.01 and *** *p* < 0.001.

To evaluate how nematode infection during colitis influenced the production of angiogenic factors in the intestine, we examined in the intestinal mucosa of mice molecules related to angiogenesis: Amphiregulin (AREG), EGF, FGF-2, FGF-7, GM-CSF, HGF, IGFBP-3, PDGF-AA, PDGF-BB, and VEGF. Colitis induction resulted in a significant reduction in GM-CSF, IGFBP-3, and both PDGF molecules compared to control mice. *H. polygyrus* infection alone resulted in an increased level of EGF, FGF-7, VEGF-A, and decreased IGFBP-3, PDGF-AA, and PDGF-BB in the intestine of mice compared to control animals. Parasitic infection during colitis was associated with elevated levels of AREG, EGF, IGFBP-3, and reduced levels of both PDGF-AA and -BB. Production of AREG, EGF, FGF-2, and IGFBP-3 was significantly higher in the intestinal mucosa of colitic mice with parasitic infection than in the intestine of mice without induced disease but infected with *H. polygyrus*. On the other hand, the levels of VEGF-A and FGF-7 were decreased in this group of mice (Figure 2).

### 3.5. Nematode Infection Influences Angiogenesis in the Brain

**Figure 3:**
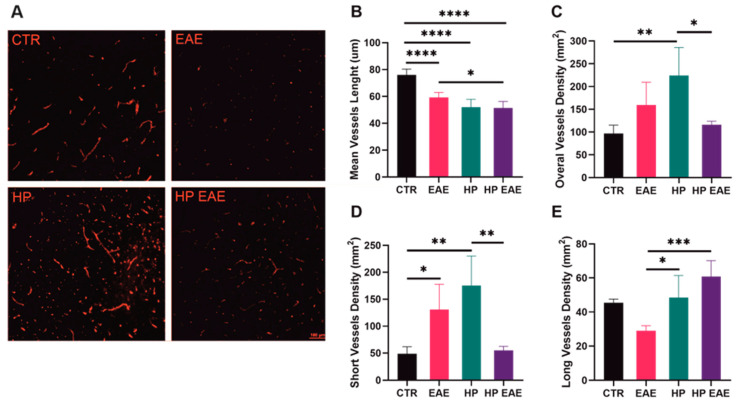
Vessels in the brains of EAE mice infected with *H. polygyrus*. C57BL/6 female mice were immunized with myelin oligodendrocyte glycoprotein (MOG35–55) 21 days before oral infection with 300 L3 *H. polygyrus*. Brain slices were stained with anti-CD31 (**A**); Mean vessel length is expressed in µm (**B**); total (**C**); short (**D**); and long (**E**) vessel densities were counted in 1 mm^2^ based on CD31 staining. Data are expressed as mean values ± SD from one representative of three independent experiments (*n* = 5 mice/group). * *p* < 0.05, ** *p* < 0.01, *** *p* < 0.001 and **** *p* < 0.0001.

To assess the effect of parasite infestation on the formation and growth of blood vessels in the brains of EAE mice, sections of the brain tissue were stained with the CD31 antibody. There were visible differences between groups in the number and density of vessels in brain tissue (Figure 3A). Mean vessel length was significantly decreased in brain tissue of EAE mice, mice infected with *H. polygyrus*, as well as EAE mice with parasitic infection in comparison to controls. In addition, vessels in EAE mice infected with *H. polygyrus* were significantly shorter than vessels in EAE mice without parasitic infection (Figure 3B). Induction of EAE in mice resulted in an increased density of short vessels. Mice infected with *H. polygyrus* had a higher density when only short vessels were analyzed as well as when the density of all vessels was taken into account. In addition, the density of long vessels in brain tissue was significantly higher in mice infected with parasites in comparison to EAE mice. Total and short vessel density in the brains of EAE mice infected with *H. polygyrus* was significantly lower than in mice infected with parasites, but without disease. In contrast, infection of EAE mice resulted in significantly higher density of long vessels compared to uninfected EAE (Figure 3C–E). 

### 3.6. EAE Induction and Parasitic Infection Influence Level of Angiogenic Factors in the Brain

**Figure 4:**
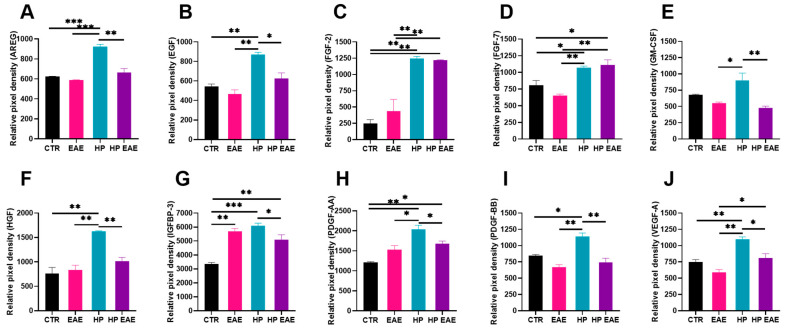
Level of angiogenic factors in cerebral spinal fluid (CSF) of C57BL/6 EAE mice infected with *H. polygyrus*. C57BL/6 female mice were immunized with myelin oligodendrocyte glycoprotein (MOG35–55) 21 days before oral infection with 300 L3 *H. polygyrus*. Mean relative pixel density of AREG (**A**), EGF (**B**), FGF-2 (**C**), FGF-7 (**D**), GM-CSF (**E**), HGF (**F**), IGFBP-3 (**G**), PDGF-AA (**H**), PDGF-BB (**I**) and VEGF-A (**J**). Data are presented as mean pixel density ± SD of two technical replicates. Data are representative for three independent experiments (*n* = 3). * *p* < 0.05, ** *p* < 0.01 and *** *p* < 0.001.

The levels of the same angiogenic factors as in the intestinal mucosa of colitic mice infected with nematode parasites were evaluated in the CSF of EAE mice with *H. polygyrus* infection. EAE development resulted in a slightly decreased level of most factors similar to colitis. However, most differences were not significant, but the level of IGFBP-3 was significantly higher in the CSF of EAE mice than of control mice (Figure 4G). *H. polygyrus* infection resulted in a significantly increased level of all evaluated growth factors: AREG, EGF, FGF-2, FGF-7, GM-CSF, HGF, IGFBP-3, PDGF-AA, PDGF-BB, and VEGF-A. Production of all molecules except FGF-2 and FGF-7 was significantly reduced in the CSF of EAE mice with parasitic infection. Levels of FGF-2 and FGF-7 were also significantly higher than in uninfected EAE mice (Figure 4C,D). 

## 4. Discussion

This study has shown a significant influence of *H. polygyrus* infection on a variety of growth factors related mainly to angiogenesis in the intestinal mucosa of C57BL/6 colitic mice and in the cerebral spinal fluid of C57BL/6 mice with experimental autoimmune encephalomyelitis:VEGF, EGF, amphiregulin (AREG), FGF-2, FGF-7, GM-CSF, HGF, IGFBP-3, PDGF-AA, PDGF-BB.

The course of EAE in mice infected with parasites and animals without infection was analogous to that observed in our previous experiments [13]. Symptoms of colitis induced with DSS in C57BL/6 mice are similar to that observed in the BALB/c strain: both strains of animals had diarrhea, considerable weight loss, and rectal bleeding [14]. However, in the C57BL/6 mice, we observed more severe symptoms of colitis compared to the BALB/c strain [14]. This is consistent with observations reported in the literature [15]. 

The effectiveness of the immune response to *H. polygyrus* differs among strains of mice. We used the C57BL/6 mouse strain, which is often used, especially in experiments on the parasite–host relationship and immunity against nematode infection. During our previous studies, we observed parasite adaptation to the inflammatory milieu in BALB/c strain mice with colitis induced by DSS. Adaptation was expressed by increased growth, survival, and reproduction, as well as a significantly higher male-to-female ratio [16]. The adaptation of *H. polygyrus* in C57BL/6 mice was reflected in the increased number of L4 as well as in the higher male-to-female ratio. The results are in line with previous observations made for BALB/c mouse strain [16]. BALB/c and C57BL/6 strains differ genetically. Those differences are reflected in various immune responses, including protection against multicellular parasites. In BALB/c there is a Th2 type immune response, but in C57BL/6 a stronger Th1 immune response is observed. In addition, BALB/c produces a stronger humoral response than C57BL/6. In our study adaption of *H. polygyrus* to the colitic microenvironment in C57BL/6 mice resulted in significant changes in cytokine levels. Localization of nematodes was similar to that observed in BALB/c mice. Larvae in control mice clustered in the duodenum, whereas larvae in mice with colitis invaded more distal regions of the small intestine (data not shown) [16]. Conversely to colitis, inflammation in the central nervous system under EAE induction in the same strain of C57BL/6 mice reduced the number of parasites compared with control infection [13]. Nematode adaptation and growth are connected with nutrition and the elimination of waste products. The formation of a network of blood vessels is an adaptation strategy [10].

Growth factors play a significant role in development of both vertebrate and invertebrate animals. Basically, they influence cell division or differentiation [17]. Growth signaling can be transmitted by various pathways and affects various biological processes. In the host, growth factors influence regeneration and wound healing [18]. Repair of the tissues frequently accompanies inflammatory and autoimmune pathology as well as multicellular parasitic infection [19]. Hence, helminths are regularly exposed to host growth factors and they have evolved appropriate responses. Helminths are strong modulators of the host milieu. Studies on the transforming growth factor beta (TGF-β) confirmed that immune response to helminths is strongly dependent on both host and parasite factors [20]. Neutralization of TGF-β affected the concentration of cytokines and their pattern of production and resulted in a reduction in worm numbers and fecal egg counts [21]. Further, nematodes produce ligands for host growth factors such as TGF-β mimics [22]. Molecular interactions between mammals and helminths are the result of the host response to the parasite, as well as parasitic activity to increase their viability and reproduction success [23]. Understanding the host–parasite relationship is important in many aspects from looking for new drugs against helminthic infection to designing new therapies based on parasite-derived products.

Angiogenesis is one of the processes involved in the pathogenesis of autoimmune diseases [24]. Capillary formation is observed in the course of multiple sclerosis. This is correlated with the release of angiogenic factors. In addition, angiogenesis has been demonstrated in EAE, an experimental model of multiple sclerosis [25]. Angiogenetic processes are important in the pathogenesis and course of inflammatory bowel diseases, with promising potential as therapeutic targets [26,27]. On the other hand, parasite infection is also associated with the influence on angiogenic processes in the host’s organism [10]. This study demonstrates the significant impact of intestinal nematode infection on growth factors related to angiogenesis in autoimmune disease attenuation.

Vascular endothelial growth factor, VEGF-A, is considered the most important regulator of angiogenesis. In our study, EAE induction resulted in increased level of VEGF-A in CSF; this is in line with previous observations in mice and multiple sclerosis patients [28]. The release of vascular permeability factor (VPF)/vascular endothelial growth factor (VEGF) is the main, although not the only, cause of the enhanced BBB permeability [29]. In EAE, astrocytes, monocytes, and activated Th1 lymphocytes all express VEGF, which leads to the disruption of the BBB [30,31]. Here, we observed the highest level of VEGF-A in the CSF during *H. polygyrus* infection, but interestingly, this level was lower when EAE mice were infected with nematodes. A similar effect of nematodes was observed in the intestinal mucosa of mice with DSS-induced colitis. Indeed, enhanced level of VEGF-A in IBD patients is associated with a worse course of the disease [32]. Helminths modulate the level of VEGF family factors. These results are in line with others. The soluble egg antigen of the liver fluke, *Schistosoma mansoni*, promotes angiogenesis by enhancing VEGF-A production by human endothelial cells [33]. The nematode *Trichinella spiralis* induces production of VEGF-A in nurse cells [34]. The free-living soil nematode *Caenorhabditis elegans* produces a PDGF/VEGF-like ligand, also called PVF1, which binds to mammalian VEGF receptors and induces angiogenesis [35]. 

The epidermal growth factor (EGF) family is also called epithelial cell-derived factors. EGF signaling is involved in angiogenesis both directly and indirectly [36]. In our study, we observed a very strong induction of EGF-pathway components during nematode infection, with even higher expressions of AREG and EGF, ligands for EGFR in mice infected with parasites and with induced colitis. Amphiregulin (AREG) is produced by many immune cells such mast cells, basophils, eosinophils, neutrophils, dendritic cells, group 2 innate lymphoid cells, and T cells. The primary role of AREG is to direct the repair damage caused by inflammation [37]. Lack of AREG in mice infected with *Trichuris muris* decreased the ability of the host to expel parasites. Lowered proliferation of colonic epithelial cells indicated a crucial role of AREG in tissue protection [38]. Our results are in line with results of Minutti [39], where infection of mice with *H. polygyrus* resulted in an increased number of CD4^+^ T cells with EGFR expression in duodenum, MLN, and spleen. EGF-pathway is important in Treg activity [40]. As a method to modulate the immune response higher levels of EGF-signaling proteins could be responsible for increased numbers of adapted nematodes. Moreover, the protozoan *Toxoplasma gondii* induces EGFR autophosphorylation to avoid the destructive autophagic process [41].

Fibroblast growth factors (FGFs) are a group of molecules involved in various developmental and metabolic processes [42]. FGF-2, also called basic FGF, is a potent mitogen and chemotactic factor for fibroblasts and endothelial cells. FGF-2 is also an angiogenic factor involved in tissue repair and wound healing [43]. FGF-2 has a protective effect on the nervous tissue [44]. We observed a significantly increased level of FGF-2 in the CSF of EAE mice infected with *H. polygyrus*. In addition, the level of FGF-2 was elevated in the intestines of colitic mice infected with the parasite, while the FGF-7 level was highest in the intestines of infected mice without induced disease. During colitis, FGF-2 signaling is essential for maintaining gut homeostasis [45]. FGF-7 ameliorates DSS colitis in mice [46]. Knowledge of the role of FGF molecules in the parasite–host relationship is very limited. Host FGF signaling influences development of the larval stage of the tapeworm *Echinococcus multilocularis* [47].

Granulocyte-macrophage colony-stimulating factor, GM-CSF, is a multifunctional factor produced by various cells, involved in immunity. GM-CSF can also influence wound healing as an angiogenesis promotor [48]. The effect of GM-CSF on autoimmunity depends on the disease entity [49]. We observed significantly increased levels of GM-CSF during nematode infection, with lower levels during inflammation. As GM-CSF seems not to be involved in the immune response against nematodes [50], participation in tissue remodeling and VEGF signaling modification may promote parasitic invasion. 

Hepatocyte growth factor (HGF) shows pleiotropic activity, including angiogenesis, by stimulating VEGF activity [51]. In our study, we observed elevated levels of HGF in the CSF of *H. polygyrus*-infected mice. So far, no studies on the involvement of HGF in the helminth–host relationship have been published. 

The Insulin-like growth factor (IGF) pathway’s major protein is IGF-binding protein 3 (IGFBP-3). IGFBP-3 is a growth factor downregulating angiogenesis [52]. In addition, upregulation of IGFBP-3 is correlated with the improvement of colitic mice, and IGFBP-3 knockout animals are resistant to DSS-induced inflammation of the colon [53,54]. Interestingly, the level of IGFBP-3 in the intestine did not change under the influence of *H. polygyrus* in healthy mice. However, IGFBP-3 was significantly increased when the parasite developed in mice with colitis. This may indicate a crucial contribution of IGFBP-3 to helminth therapy. IGFBP-3 in CSF is highest in mice infected with *H. polygyrus*.

The PDGF growth factor family controls proliferation, differentiation, chemotaxis, and angiogenesis and is involved in wound healing. A low level of PDGF-AA and -BB was observed in the intestines of mice infected with *H. polygyrus*, with an even lower level in colitis mice with parasitic infection. Elevated levels of PDGFs are associated with a worse course of inflammatory bowel diseases [55]. On the other hand, the highest level of PDGF factors in CSF was observed in mice infected with nematodes. This is in line with observations that higher production of PDGF molecules in human CSF correlates with anti-inflammatory microenvironment during multiple sclerosis [56].

In our study, we also observed significant differences in vessels in EAE mice brains. The shortest vessels were observed in EAE mice infected with parasites. In contrast, in this group of mice, the density of long vessels was the highest. This observation suggests that angiogenesis in helminth therapy proceeds in a different way. Although this hypothesis needs to be confirmed, future therapeutic efforts against MS should perhaps focus more on developing new vasculature with functional integration and less on total angiogenesis blockade [25].

## 5. Conclusions

Intestinal nematode infection altered the angiogenic growth factor response. There was an increase in VEGF, EGF, amphiregulin (AREG), FGF-2, FGF-7, GM-CSF, HGF, IGFBP-3, PDGF-AA, and PDGF-BB. The parasite’s promotion of growth factors related to angiogenesis increases vascular permeability and could inhibit the host’s immunological response and assist helminth survival. Parasitic nematodes are very well adapted, hence their influence on growth factors is not surprising. Interestingly, nematode infection altered the growth factor response in inflammatory conditions, such as in murine colitis and multiple sclerosis. Although more research is needed, nematode-derived growth factors appear to be promising tools to control autoimmune diseases. 

## Figures and Tables

**Table 1:** Level of cytokines in serum, colon, and small intestine of C57BL/6 mice with induced colitis and parasitic infection. C57BL/6 male mice were given 3% dextran sulphate sodium (DSS) in drinking water for two days before being infected orally with 300 L3 *H. polygyrus*. Levels of IL-1β, IL-6, TNF-α, IL-10, and TGF-β were measured by ELISA. HP—mice infected with *H. polygyrus*; HP COL—colitis mice infected with *H. polygyrus*. Data are shown as means ± SD from one of three independent experiments (*n* = 3–5 mice/group).

Cytokine		Serum	Colon	Small Intestine
IL-1β	HP	2.9 ± 1.1	154.5 ± 54	113.5 ± 64
	HP COL	7.5 ± 7.5	2518 ± 638 *	144.3 ± 38.4
IL-6	HP	1.2 ± 2.8	206.9 ± 66.2	not detected
	HP COL	241 ± 363.1 *	360.3 ± 89.7 *	150 ± 120.1 *
TNF-α	HP	28.8 ± 4.5	148.5 ± 40.2	222.6 ± 144.8
	HP COL	73.4 ± 22.2 *	130.7 ± 32	921 ± 608.3
IL-10	HP	3685.1 ± 1829.8	8973.6 ± 3336.7	4854.2 ± 1362
	HP COL	6735.6 ± 2101.2 *	9833.1 ± 3740.8	5471.3 ± 2256.6
TGF-β	HP	977.7 ± 418.4	19.7 ± 18.5	4655 ± 1267.7
	HP COL	479.7 ± 167	38 ± 22.7	5099.9 ± 4049.9

* *p* < 0.05 compared to HP; pg/mL; Mean ± SD; Student’s *t*-test or Mann–Whitney test.

## Data Availability

Not applicable.
